# Hospital and Institutional News

**Published:** 1913-02-01

**Authors:** 


### HOSPITAL AND INSTITUTIONAL NEWS.

### A GOVERNMENT CANVASS FOR A PANEL DOCTOR.

The sanction which the Insurance officials have given to the action of the Hearts of Oak members at Kingston in actively canvassing for a Welsh doctor, who was imported as a "blackleg" some time ago, because no local practitioner could be induced to serve the members, is likely to increase the bitterness already rife among patients and doctors by means of the panel system. Last year the medical officer to the local Hearts of Oak Medical Society resigned, and his successor was supported by the local branch of the British Medical Association in refusing to accept a number of the older members. The Society objected, but the Association stood firm, and, as explained, a medical boycott of the Society occurred in the neighbourhood. The Welsh "blackleg," with the Society's aid, started a practice at Kingston, and an active canvass is now being made by his backers to obtain two thousand insured patients for him. On the legality of this action being questioned, the Insurance officials have replied that there is nothing illegal in the practice, as the doctor is on the panel, and if two thousand insured persons choose to select him they have a perfect right to do so. Quite so; but if they did choose, there would be no need to canvass. The action of the Canvassing Society is plainly open to grave abuse, and if not repudiated by the Commissioners may lead in the present condition of affairs to "peaceful picketing" of the patients and to tyranny over them. Would it not amount, in effect, to a Government canvass?

### DROPPING THE PANEL.

At Wisbech and Chatteris, where the panels have proved inadequate, the Insurance Commissioners have decided to dispense with the panel system, and the Isle of Ely Insurance Committee has been authorised to make arrangements for outside medical men to attend insured persons for the present. The Chairman of the Committee added that such arrangements would be without limitation in the matter of private practice. In the end it was decided to offer the appointment for Wisbech to Dr. Horace Dimock, of Ely, and the appointment for Chatteris to Dr. Coffey.

### WHERE IS FORM "MED. 21"?

Where is Form "Med. 21"? Dr. Ernest W. Lowry, of Kew Bridge, is quite right to press this question in view of the difficulties which are being thrown in the way of insured persons who desire to exercise their right freely to choose a doctor who does not happen to be upon the panel. "It is a singular commentary on the action of the Insurance authorities that Form 'Med. 21,' which has been printed and exists at the head office of the Insurance Commisioners, should have been withheld from the public. This form is headed, "Form of notice by person desiring (or required) to make his own arrangements for treatment." "This form makes provision for two definite classes of persons contracting out—those who desire to enter into a contract with a non-panel doctor at a fixed sum per annum; and those who desire to be treated as private patients upon non-contract terms. These latter are instructed to send in the accounts received by them from the doctors from whom they have obtained medical treatment." Of course it is obvious that the moment the majority of insured persons realise that they have, in fact, been deprived of a free choice of doctor, the demand for Form "Med. 21" will be very brisk. Any action which may amount to its continued withholding from the public must be carefully watched, until form "Med. 21" and its provisions become as well known to the public as a certain Form 4 became not long ago.

### THE FIGHT FOR FREE CHOICE OF DOCTOR.

A question which is now exercising the minds of doctors who are not on the panel and of patients independent enough to make a fight for that freedom of choice in the matter of doctors which fast seems to be eluding them is this: To what allowance will an insured person be entitled who demands to be attended by a practitioner who is not on the panel? Unless the Insurance Committees are deliberately trying to escape from their obligations by rejecting as unsuitable all arrangements with non-panel doctors, irrespectively, Dr. H. J. Starling, of Norwich, points out that Regulation 49, Section 2, and the Commissioners' Explanatory Statement issued in December last, Section 6, make it plain that the insured person who makes his own medical arrangement is entitled within limits to the cost of the treatment. All who have cause to fight for free choice of doctor, and it is one which patients and the medical profession have an equal interest in maintaining at all costs, should study the clauses enumerated above, together with Section 44 of the Explanatory Statement referred to, which actually goes into details as to how the doctors' bills are to be paid.

### DESICCATED MILK FOR INSTITUTIONAL USE.

Our Special Commissioner, who has been investigating the economic values of hospital foodstuffs, would be grateful if hospital secretaries will kindly furnish him with particulars regarding the extent and manner in which desiccated milk is used in their institutions. The points on which he specially desires information are these: (1) What brand of desiccated milk is used? (2) What percentage of cream does it contain? (3) What price, wholesale, is paid for it per hundredweight, and how does this price compare with the price of ordinary dairy milk in your district? (4) How is the desiccated milk prepared for ward or kitchen consumption—we should like to know, for instance, whether the institutional milk pans are of aluminium or nickel? and, finally, (5) How does the milk so produced compare, in quality and taste, with ordinary dairy milk? We venture to think that this investigation, the results of which we hope to publish in the columns of The Hospital, will be of practical interest to many institutional workers. The ordinary price of dairy milk in London varies from 3½d. to 4d. per quart retail; desiccated milk, properly prepared, and dietetically equal to dairy milk, averages 2d. per quart. The difference in price is so great that institutional workers can no longer neglect this new foodstuff, the economic value of which has been proved by experimental trial in America and on the Continent. It is somewhat difficult to obtain good brands of desiccated milk, especially in large quantities, and we do not yet know what the institutional experience is regarding its value. For that reason we shall be grateful if hospital secretaries and others, who have had any practical experience of desiccated milk, will kindly communicate with us and answer, briefly, the questions outlined above.

### THE GRAND DUKE MICHAEL'S GIFT.

The first ambulance of its kind to be used outside the City has been presented to the Hampstead and North-West London Hospital by the Grand Duke Michael. At least, his gift has taken the form of a cheque for five hundred guineas for the purchase of a motor ambulance of a similar type to that now used in the City of London. The offer has been gratefully accepted, and the London County Council has been approached with a request to provide the £200 a year which is required for its maintenance. Should any difficulties be experienced we hope that a careful record of its first year's work will be kept, so that the hospital's experience of it, both in respect of its value and drawbacks—for everything has drawbacks—may be published for the benefit of other institutions and for the encouragement of the subscribers of the first hospital to adopt an ambulance of this kind.

### THE DOCTOR AND GENERAL ACCOMPLISHMENTS.

"Your recent allusion to the affinity between poetry and medicine," writes a correspondent, "is one which not a few Bristol medical men are likely to confirm who call to mind the poetical gifts of their former citizen, the late Dr. John Addington Symonds, author of various pieces of verse, and renderings also in verse from the Greek Anthology. When, fifty years ago, Dr. Symonds delivered his President's Address at the meeting of the British Medical Association in Bristol, 1863, he said: 'It is but too true that once the general accomplishments of the medical practitioner were regarded with suspicion, as if that man could not be trusted in the exercise of his art who made his recreations and embellished his life by the study of polite letters and fine arts. In those times he might mount his hunter or sling his fowling-piece over his shoulder, or walk to the bedside in the attire of a sportsman, but if he were suspected of an ode or a sonnet the jeopardy (to his reputation) was extreme! Happily we live in wiser and better times.'" Indeed, the number of books published as popular versions of technical subjects, apart from the popular light essays in a medical vein, leads critics to say that no qualifications are held by the public complete unless the word "publications" in the stock medical reference works is followed by a list of books, pamphlets, or at least newspaper articles.

### A CURIOUS POISONING CASE.

A recent case at the Berkshire Assizes is a notable example of the tendency of the modern jury to award heavy damages to suitors whose injuries have been nominal or at least not grievous. A mother at Hungerford asked her next-door neighbour, a chemist, to supply some glycerine suppositories for her baby. By a mistake, morphia suppositories were supplied instead; it is not quite clear whether the mistake was the fault of the chemist in selling a wrong packet, or of the wholesale manufacturer in wrongly labelling the packet. At any rate, the baby soon exhibited symptoms of acute morphine poisoning, and for a day or two was in a critical condition. Its life was, however, eventually saved by energetic and prolonged treatment. No after-effects of any moment were alleged, but an action was begun by the parents on behalf of the baby. The jury awarded £50 damages, besides the doctor's fees and other expenses, notwithstanding a pronouncement from the Bench which showed that the Judge was in favour of a nominal award. Since the baby was much too young to have any recollection of the affair, and, in any case, morphine poisoning cannot be described as a painful accident, and no ill-effects persisted, it seems that the allotted sum is excessive; probably the jurymen were influenced by those feelings of class sympathy which seem frequently to actuate juries nowadays.

### "RECHRISTENING" THE RED CROSS.

At the recent banquet offered by the Bulgarian Red Cross Society to Surgeon-General Bourke, of the British Red Cross Mission, a well-informed foreign correspondent states that the Red Cross and Red Crescent symbols were objected to. The objection was made on the concrete ground that in a war to which Mahomedans were a party both were assumed to possess a religious significance by the more ignorant peasants, with the result of severely hampering the relief work. Only a person who has worked on the spot can tell the extent to which this objection is a real one. In the meantime, it would not be so easy to suggest a substitute that will have equal prestige without similar association. We invite our readers' criticisms of this sweeping suggestion.

### COMPLAINTS AND THE SATURDAY FUND.

For the first time since 1898 there has been a reduction, amounting to £2,000, in the sum collected by the Hospital Saturday Fund. Grants amounting to £32,305 are proposed by the Committee. The motion to approve these awards, however, led to suggestions that subscribers to the Fund were being refused treatment at the hospitals, and were being handed over to panel practitioners. An amendment, asking the institutions to guarantee to treat the Fund's patients before receiving its grant, having been moved and seconded by Mr. Summers and Mr. Clark, was successfully disposed of by Mr. A. W. Davis, the secretary, who urged a waiting policy till the Special Committee already appointed on this question shall have presented its report early this month. Mr. Davis added that ninety-nine out of every hundred complaints which he had received should never have been made. The common-sense value of his remarks was endorsed by the large majority which defeated the amendment.

### FURTHER FRUITS OF PANEL SERVITUDE.

An inquest was held at Enfield last Saturday on a domestic servant, who had been attended on the day that she was taken ill by Dr. Distin, when he was unable to diagnose her condition. He prescribed and said he would call the next day but one. The day following, however, he was sent for, but owing to the fact that he had over one hundred callers asking for their cards to be signed he was prevented from attending. Later he was informed that the girl was dead. Mr. A. M. M. Forbes, the Coroner, said that owing to the very heavy clerical work under the Insurance Act the doctor had not been able to give proper medical attendance. A verdict of natural death, apparently heart-failure resulting from apical pneumonia, was returned.

### THE CONFINEMENT OF CONSUMPTIVES.

There are some observations so profound as never to become trite. One of them is that human nature is the same everywhere. When Peamount Sanatorium, near Dublin, was begun, the local peasants wrecked it. When it is proposed to treat Insurance Act consumptives at the Downs school buildings at Sutton, the District Council puts up a doctor to speak against the scheme. It is probably not fear of infection which is the real motive, but a rankling sense of having been victimised, or the apprehension of debile and shabby-looking patients strolling about the place and frightening away prosperous residents. This is one of the things those interested in institutional work have to put up with. From the reply of the Chairman of the Metropolitan Asylum District Managers to a deputation it would seem as if the consent of all necessary bodies, including the Local Government Board, having been secured, there is no chance of anything being altered; and this being so, our part is to give the Sutton residents all the consolation we can. But Dr. Hooper did not make a good point when he claimed that at Sutton the patients could not be " kept within the four walls of the building." It would be very poor sort of open-air treatment if they were. But it may be admitted that consumptives should be confined to grounds, where these are large enough, and only allowed out on public ground under strict precautions. These buildings, not erected expressly for sanatorium treatment, do not make proper sanatorium discipline quite easy, and the Metropolitan Asylums Board will be well advised to choose for their medical superintendent in such cases a conscientious man with plenty of special experience. Local objections, however, have a way of dying out. At a public sanatorium within our knowledge the neighbours came and threw tar over the newly-painted entrance gates; but within a year or two, when it was seen that the institution was spending nearly £2,000 a year in the district on meat, coal, groceries, and milk, they realised that the sanatorium was one of the mainstays of the place and worth a good many country houses! Matters may be a little different at Sutton, but 300 immigrants into a locality cannot fail to bring some custom, and the example of a sanatorium, Mount Vernon, at another rich and eligible London suburb, Northwood, shows beyond any dispute that a well-managed institution of this kind is no nuisance or danger at all.

### DOCTOR'S ACTION AGAINST A FRIENDLY SOCIETY.

The action of Dr. H. J. Twamley, of Sible Hedingham, in suing a member of the Castle Hedingham Branch of the United Patriots Benefit Society, to which he is medical officer, for five guineas for medical treatment, which the doctor lost, has a point or two of special interest. The defendant, it appears, met with an accident, and was attended by Dr. Twamley in Halstead Cottage Hospital, a place which the doctor now stated as regarded by him as outside his district. He claimed the defendant, therefore, as an ordinary patient. The defendant contended that he was a club patient, and that, though the accident happened outside the district, he was still residing within its boundaries. A reference to the rules elicited the fact that the club's doctors were to attend " all members residing in their vicinity "—a horribly lax phrase, which we are glad to note that Judge Tindal-Atkinson condemned while giving judgment against the doctor.

### THE NEW HOUNSLOW HOSPITAL DESIGN.

In Staines Road, Hounslow, a new hospital has been erected from designs by Mr. J. Ernest Franck, A.R.I.B.A., M.R.San.I. Opened last week, it provides, in place of the original hospital, which had seventeen beds, two ten-bed wards and two two-bed wards; twelve beds are allocated respectively to male and female cases. The long axis of the wards is north-west and south-east, and disconnected sanitary blocks are placed at the south end of each main ward. A central entrance to the centrally placed administrative block gives access to the committee, matron's, and consulting rooms, while the dispensary patients have a separate entrance on the west side. The operating room and its adjuncts are placed in a projecting block to the north, opposite the entrance, and they are connected by corridor to the wards on each side. A feature of the plan is the situation of the kitchen and offices. These are placed in a wing at the south-east corner; this has probably developed by the architect's desire to get the operating room central to both wings, but is open to criticism, for whereas operations are not necessarily of everyday occurrence, the food question is one to be faced three or four times in each twenty-four hours, and, therefore, should have first consideration. The administrative block is two storeys high, and the second floor contains staff bedrooms; provision has been made for another storey to be added to the wards should this be found necessary in the future. The elevations are treated in a quiet and restrained manner, with stock bricks, stone dressings, and slated roof.

### THE SCOTCH COTTAGE HOSPITAL TO-DAY.

Our note last week on a Scotch Cottage Hospital Movement has elicited a prompt reply from the Matron of the West Highland Cottage Hospital, Oban. She encloses an interesting report, but, while apparently deprecating the criticisms made by the Committee appointed by the Treasury to investigate Medical Service in the Highlands, does not discuss the general charges which we summarised. To say that the semi-emptiness of the cottage hospitals in the Highlands was due to bad management, or financial reasons, was certainly a sweeping statement which ought not to be allowed to pass without an explanation or reply from the institutions on which it reflects. The Gesto Hospital, Edinbane, Isle of Skye, and the Dunbar Hospital were mentioned specifically either as being in some difficulty or as not fully serving the town and district. What is the position of these hospitals in regard to the Treasury Committee's Report? Dr. Currie, the medical superintendent of the hospital whose Matron writes this week, and Mr. John Sutherland, the hon. secretary, should be able to throw some light on the true factors of the case. We shall be very glad to publish criticisms of the Report from any of the institutions affected.

### NEW PROFESSOR OF TROPICAL MEDICINE.

Dr. J. W. W. Stephens has been appointed to the Sir Alfred Jones Chair of Tropical Medicine at the University of Liverpool, in succession to Sir Ronald Ross, who, as we noted recently, has come to take up work in London. Dr. Stephens graduated in medicine at Cambridge, and has held the Walter Myers Lectureship in Tropical Medicine, besides teaching at the Liverpool School for some ten years past.

### A HOSPITAL CHAPLAIN AND HIS CHAPEL.

The death is announced of the Rev. Arthur Brinckman on January 28 at St. Saviour's Hospital, Osnaburgh Street, N.W. Mr. Brinckman's career was as varied and interesting as that of the chapel in which his ministrations were conducted. For five years he was in the 94th Regiment, from 1855 to 1860. Then he gave up the Army in order to take Orders in the Church of England, into which he was ordained fifty years ago, in 1863. After holding a curacy in Berkshire he was mission priest in Edinburgh, and then for seventeen years was curate at All Saints, Margaret Street, well known for its ritualistic congregation and clergy. Some twenty years ago he was appointed chaplain of St. Saviour's Hospital, with which he combined the care of St. Agnes Chapel, Sutherland Avenue. Mr. Brinckman's chapel at Osnaburgh Street is one of the sights of London, though there are perhaps very few of the general public who are aware of its existence or its beauties. The whole of the interior is constructed of mediæval carved woodwork of magnificent workmanship. This was executed in the seventeenth century in the Low Countries by monks; it was purchased early in the last century and brought over to England. The venerable lady who presides over the management of St. Saviour's, Sister Caroline Mary, takes the greatest care of this lovely chapel and is never happier than when showing it to some favoured visitor. At some future date we hope to give an account of the special work which is done so well and yet so unassumingly at this institution.

### THE PROPRIETARY MEDICINES INQUIRY.

Some interesting evidence has been heard by the Select Committee on Proprietary Medicines since the inquiry was resumed, the principal witness being Sir Joseph Beecham, who appeared not only as representing his own business but as Chairman of the Proprietary Articles Section of the London Chamber of Commerce. An idea of the enormous output of proprietary remedies can be obtained from the statement made by this witness that over a million Beecham's pills are sold daily, while over £100,000 was spent in advertising them last year. "Secret Remedies" gives the composition of Beecham's pills as aloes, ginger, and soap, but Sir Joseph said there were important ingredients which the analyst had not found. With regard to Beecham's cough pills "Secret Remedies" says that, in spite of the printed statement that the pills do not contain opium, analysis shows them to contain morphine. Asked to explain this, Sir Joseph said the circular in which this statement appeared was withdrawn about five years ago, having been printed during the period when the pills did not contain morphine. It appears that when the cough pills were first introduced, some sixty years ago, one of the ingredients was morphine, but this drug was subsequently eliminated, because with that ingredient the pills could not be sold by grocers. At a later period, however, a small quantity of morphine was again added to the pills. These facts having been stated, the Chairman suggested that it would appear that the medicinal ingredients of the pills were dependent, not upon scientific or medical knowledge, but upon certain requirements of the law at the time, but Sir Joseph would not agree to this, his explanation being that the pills were quite good without morphine. It is an interesting point, and no doubt the Committee sees the importance of it.

### THE ENDOWMENT OF A THEORY.

The British Homœopathic Association has received £5,000 from Mr. Otto Beit as a fund the proceeds of which are to be devoted to research into the problems of medicine, particularly into those the solution of which is likely to throw light upon the range and mode of action of the homœopathic law. It is a refreshing change to find a layman assisting to endow a theory of the cure of disease rather than a process of treatment; and, indeed, to such an one sincerely anxious to devote his money for the true advancement of medical knowledge, it is only natural that those who proceed from a general theory of some kind, whether mistaken or even only half true, should command his sympathetic interest. The *a posteriori* method of inquiry is one which has established its value beyond criticism, but, as we are beginning to find, it seems hopeless of advancing medical science as a whole so long as it confines itself to experiments in the sphere of treatment, while passing by as unworthy of attention all *a priori* theories of disease and of the principles of its cure.

### AN EDINBURGH HOSPITAL PERSONALITY.

Whether it is the University atmosphere or something innate in the Scottish character, at all events it is true that outstanding personalities are, and have always been, plentiful in the Edinburgh medical school. The late Dr. G. A. Gibson, who died last week, was a notable example in point. It was not only that he was an eminent physician and a brilliant member of the medical staff at the Edinburgh Infirmary; he was, beyond that, a man of rare character and lofty ideals, and a really scientific clinician of the most progressive and practical type. It is remarkable to note that, although he qualified in 1876 and from the first devoted himself to research and teaching as preparation for a career as a consultant, it was not until 1890 that he became assistant physician on the staff of the Royal Infirmary. When this appointment was his, he was fortunate in being attached to Sir T. Grainger Stewart, who practically gave him the run of his wards; thus Gibson got in the end ample opportunities of clinical study, even though he had to wait some years for them. He devoted himself almost from the first to diseases of the heart, and was one of the first to appreciate the new epoch in cardiac medicine created by Mackenzie and his followers. Dr. Gibson had also lately been appointed representative of the Royal College of Physicians, Edinburgh, on the General Medical Council. He was a man who will be much missed both in the medical school and hospital world of Edinburgh.

### THE NEWCASTLE INFIRMARY CHAPEL DISPUTE.

At the time of going to press Lord Justice Farwell has not given his reserved judgment in what everyone must hope will prove the final settlement of the long dispute which led to the closing of the chapel at the Royal Victoria Infirmary, Newcastle. We have chronicled each bitter stage in the long controversy as to whether, according to the statutes and rules of the institution, the house committee has power to devote the new chapel to the celebration of Church of England services alone. In order to explain how matters have reached the cognisance of the Chancery Division of the High Court, we must remind our readers that towards the end of October 1910 a court of the infirmary governors—which was held to consider the refusal of the house committee to act on the governors' instructions and to open the chapel on equal terms to Church of England and Nonconformist services —decided to adopt a pacific suggestion of the then Lord Mayor. His proposal was that a committee representing the governors be appointed to instruct two firms of solicitors—the one for the governing body, the other for the Nonconformists. These solicitors should then draw up a plain statement of fact, and make a joint appeal to the High Court to see if the original consecration of the chapel, according to the rites of the Church of England solely, had been legal or illegal. It should be added that the then Lord Mayor generously promised to bear the cost of the proceedings himself. We must now thankfully wait, making no comment, till Lord Justice Farwell gives his judgment.

### PATIENTS' DIET IN AN ISOLATION HOSPITAL.

We alluded last month to the suggestion of Mr. W. Fletcher, a member of the Wolverhampton Board of Guardians, that a committee should be appointed to draw up a dietary table for the patients at Moxley, the converted smallpox hospital of the South Staffordshire Joint Smallpox Hospital Board. At a meeting last week, which succeeded an inspection, Mr. Fletcher said that all the patients had expressed their satisfaction with their diet and treatment. "They are given," he said, "a breakfast of porridge, bacon, and two mornings a week ham; at eleven o'clock half a pint of milk; a dinner of meat, two kinds of vegetables, and pudding; and tea of bread and butter, marmalade, tomatoes, and celery; and supper of cocoa or soup, biscuits and cheese." Treatment by committee, to which, in fact, Mr. Fletcher's suggestion amounted, could hardly do better than this, and Dr. Lee, the resident medical officer, must have regarded its recital as an *amende honorable!* We trust that the day will never come when proposals for removing treatment out of individual hands will be listened to for a moment. But the actual dietary in use at Moxley is an interesting example of the standard obtaining in converted isolation hospitals at the present time.

### THE WORKPEOPLE'S FUND AT SCARBOROUGH.

From having continually followed with interest the growth and multiplication of Workpeople's Funds we are sorry to record a serious falling-off in that at Scarborough. It has fallen for the first time in its history, and the decrease is virtually one-third. The Scarborough Workpeople's Hospital Fund, moreover, has made great efforts to encourage collectors by the institution of a medal, while the presentation of a shield is the reward of the firm which has achieved the best result. The committee shows no sign of discouragement in its report, however, and in this way presents an example of keenness worthy of its cause. Mr. G. Taylor, the honorary secretary of the fund, who presented this report, also outlined a scheme for raising a fund to give special assistance to persons unaffected by the Insurance Act who required convalescent treatment. Mr. E. J. Crome, the fund's representative on the hospital board, denied the strange rumour that the families of insured persons had been refused treatment as outdoor patients. He pointed out that the hospital treated many uninsured persons in the town, particularly the dependants of insured workers.

### WOMEN AS SCHOOL MEDICAL OFFICERS.

To fill the vacancy caused by the resignation of Dr. Lilian Wilson the Acton Education Committee have now appointed Dr. Elsie Mary Chubb, of Tunbridge Wells, lady assistant school medical officer and assistant medical officer of health. She graduated B.A. at the Cape of Good Hope, and was awarded the Porter Scholarship of £200 a year for three years; and is also M.D. London and D.H.P. Dr. Chubb has had experience of work amongst children, having held appointments as obstetric assistant at the New Hospital for Women, Euston Road, and house physician and house surgeon at the Victoria Hospital for Children, Hull, and in regard to the training of midwives for maternity work. She holds at present the Helen Prideaux Scholarship, which is awarded every two years by the London School of Medicine for Women. The course of study now being undertaken includes work at the Königliche Charité, Berlin. Dr. Dora Elizabeth Lidgett Bunting, of Torrington Place, London, was the other selected candidate, but subsequently withdrew her application.

### A PUBLIC ANALYST AND SUPERANNUATION.

The Camberwell Borough Council has considered the question of deducting superannuation contributions from extra payments over £400 per annum —in respect of special samples and Court fees— made to Dr. F. L. Teed, D.Sc., public analyst for the borough. The important points are contained in two sections: Section 13 of the Superannuation Act provides for the percentage amounts to be deducted from each and every payment made to officers and servants by way of salary or emoluments; while Section 3 takes care to explain that the expression "emoluments" includes all fees, poundage, and other payments made to any officer or servant as such for his own use by the Council. The Council has, therefore, decided to instruct the Borough Treasurer to make deductions from all payments made by the Council to Dr. Teed in future.

### THE PRESENT POSITION OF DISPENSERS.

This week and last several questions were asked in the House of Commons by Mr. Touche and other members who are anxious that dispensers who do not possess the pharmaceutical diploma should not be thrown out of employment as a result of the operation of the Insurance Act. The object of these questions was to ascertain under what conditions dispensers could continue in their employment without infringing the Act; while it is clear from the answers that there may be a few cases of hardship. The effect of the wholesale transfer of dispensing from doctors to pharmacists has undoubtedly been to diminish the number of dispensers employed by medical practitioners, but there is no reason whatever why displaced dispensers should not enter the service of pharmacists. As a matter of fact there is in several important centres a dearth of chemists' assistants, and pharmacists are anxious to secure the services of those who have been accustomed to dispense medicines. The position of dispensers was fully explained in The Hospital in June last, and nothing has occurred since those explanatory articles appeared to alter their position. In point of fact it may be urged that dispensers are better off.

### FROM DISPENSER TO PHARMACIST AT DALSTON.

The January pass-list of the Pharmaceutical Society includes the name of Mr. Albert Howell, who qualifies as a pharmacist at his first attempt. Mr. Howell has been dispenser at the Dalston Dispensary for many years, and as hon. secretary of the Certified Dispensers' Association has taken an active part in attempts to raise the status of the "apothecaries' assistant." This Association has pressed, unsuccessfully, for inclusion on the Chemists' and Druggists' Register of dispensers certified under the Apothecaries Act, who have been in responsible positions for six years, without further examination. To the ordinary young aspirant the requirements of the Pharmaceutical examination prove sufficiently difficult, therefore we remark upon Mr. Howell's success with pleasure. He has shown a very practical way out of the restrictions of the dispenser who has stopped too soon in his educational career.

### THIS WEEK'S DRUG MARKET.

The improvement in business noticed last week has been maintained, wholesale druggists having been especially busy in consequence of the increased demand for drugs caused by the coming into operation of the medical benefit under the Insurance Act. As was anticipated, makers of quinine have added one penny per ounce to their prices, but as yet no official announcment has been made concerning the reported agreement between the growers of cinchona-bark and the quinine manufacturers. If and when the agreement is signed a further advance in the price of quinine will probably take place. Citric acid is dearer, and in consequence there has been an advance in the prices of potassium citrate, sodium citrate, and iron and ammonium citrate; while there has been a double advance in the quotations for iron and quinine citrate. High prices are asked for ergot of rye. Sarsaparilla is somewhat dearer. There has been a further rise in the value of essence of lemon, and the price of oil of orange has also advanced.

### TO OUR READERS.

Contributions are specially invited from any of our readers to these columns. They should deal with topical subjects and news. They must be authenticated for the information of the Editor only. The minimum payment if published is 5s. There is no hard-and-fast rule as to space, but notes of about twenty lines in length are preferred.

